# Multiple inositol polyphosphate phosphatase: a hidden phytate digester with bioactive function potential in animal husbandry: review

**DOI:** 10.5713/ab.25.0122

**Published:** 2025-06-10

**Authors:** Jaiesoon Cho

**Affiliations:** 1 Department of Animal Science and Technology, Konkuk University, Seoul, Korea

**Keywords:** Farm Animals, Multiple Inositol Polyphosphate Phosphatase, Physiological Events, Phytase, Phytate

## Abstract

The objective of this review was to describe the enzymatic properties of multiple inositol polyphosphate phosphatase (MINPP1/MIPP) as an unusual member of histidine acid phosphatase, distinct from conventional microbial phytases and their additional physiological functions besides degrading phytate. Considering parameters such as pH activity profile, substrate specificity, catalytic efficiency, and stability, MINPP1 is of merit as a novel phytase source for developing an ideal feed additive supported by functional metagenomics fused with recombinant DNA technology and classical protein engineering. In addition, MINPP1 appears to be involved in some biological activities such as cell survival, stress, lipopolysaccharide and inorganic polyphosphate-induced inflammatory response, milk fatty acid composition-related metabolism and bone-related growth and pathophysiology, which can be important for the production performance of farm animals. Future directions need profound studies revealing the direct effects of MINPP1 on these physiological events.

## INTRODUCTION

Up to now, phytase has remarkably contributed to the improvement of bioavailability of phosphorus and other nutrients such as minerals and protein in sectors of swine nutrition, poultry nutrition, and aquaculture, solving issues of environmental phosphorus pollution [1–3]. In the global enzyme market, the business of phytase is still a hot spot. It has been estimated that the phytase market size will be grown to 624 million USD by the year 2026 [4]. Commercial phytases have been largely established from bacterial and fungal sources [4].

Some studies have investigated biological functions of phytase beyond the nutritional aspect to dephosphorylate phytate, also named as inositol hexaphosphate (InsP_6_) [5–16]. For instance, supplemental phytase can affect the taxonomic profile of bacterial metagenome in the ceca of growing pigs [13] and change plasma metabolome profile in broiler chickens [14], which might be associated with host physiology and health. Phytase also appears to improve the immune response in fish [15,16]. However, details of these mechanisms remain unclear and poorly understood (Table 1).

Ongoing study concerning finding new bioactive phytase sourses with potential action of physiology as well as desirable catalytic efficiency of phytate is worthwhile to open up a new market in the existing feed industry. In this regard, multiple inositol polyphosphate phosphatase (MINPP1/MIPP) can be a good alternative (Table 2). The catalytic function of MINPP1 has been mainly reported from animal and plant [17–19]. However, it has been recently identified from prokaryotes such as gut and soil bacteria [20–23]. Although it is not well known, this unusual enzyme can be categorized into a subgroup of histidine acid phosphatase (HAP) family, to which commercially representative microbial phytases such as *Escherichia coli* and *Aspergillus* sp. phytases belong [23,24]. Interestingly, MINPP1 has been reported to be associated with a variety of physiological events [18,25–34]. This short review concentrates on the enzymatic evaluation of MINPP1 as a digester of phytate and its potential bioactive functions that have never been expected, which might be effectively applied in animal husbandry.

## BIOCHEMICAL CHARACTERIZATION OF MINPP1

### Motif analysis

Based on protein structures, microbial phytases of the HAP family are typically characterized by two conserved motifs such as the active-site heptapeptide, Arg-His-Gly-x-Arg-x-Pro (RHGxRxP) and the proton donor dipeptide, His-Asp (HD) [24,35]. However, the proton donor sequence in avian, human, wheat, barley, *Bacteroides thetaiotaomicron* (Bt), *Acinetobacter* sp. (AC 1-2), *Bifidobacterium pseudocatenulatum* (PhypA) and *Bifidobacterium longum* subsp*. infantis* (PhylA) MINPP1 is replaced by a tripeptide, His-Ala-Glu (HAE) [17–23]. AC 1-2, phypA and phylA MINPP1 have the modified heptapeptide active-site motif, Arg-His-Gly-Ser-Arg-Gly-Leu (RHGSRGL), instead of the RHGxRxP [21–23]. In addition, eukaryotic MINPP1s contain a typical Lys-Asp-Glu-Leu (KDEL)-like endoplasmic reticulum (ER) retention tetrapeptide sequence at their C-terminal ends [36]. SDEL and ADEL sequences are present in human and avian, respectively, while KT(Thr)EL is present in plants including barley and wheat [17,19]. In this regard, Yu et al [37] mentioned about the importance of MINPP1’s confinement to the ER, emphasizing that inositol lipid signaling and inositol phosphate metabolism can be independently regulated.

### Optimum pH

In terms of the pH activity profile, microbial phytases of the HAP family work very well at acidic pH range of 2.5–5.5 with one or bi-hump pH optima [38–40]. Their activities are almost inactivated at pH 6 [38–40]. Even if plant (barley and wheat) and *Bifidobacterium* (phypA and phylA) MINPP1 show their single pH optima of 4.5 and 5.5, respectively [19,22,23], bacterial MINPP1 including Bt and AC 1–2 possess multiple pH optima [20,21]. Avian, Bt and AC 1–2 MINPP1 are also effective at pH 7.4 [17,20,21]. Such a relatively broad pH range of the activity in these enzymes will be of merit for applying them to gastrointestinal tracts of farm animals with variable pH spectra.

### Enzyme kinetics

Considering kinetic parameters of three representative microbial phytases, the Km value (μM) indicating the substrate affinity was 660–1,030 for phytate in *E. coli*, 138–4,700 for phytate in *Bacillus* sp., and 27–124 for phytate in *Aspergillus ficuum* (*niger*) [38–41]. The Vmax value (μmol/min/mg) indicating the maximal reaction velocity was 89–117 for phytate in *E. coli*, 1.32–49.01 for phytate in *Bacillus* sp., and 180–216 for phytate in *Aspergillus ficuum* (*niger*) [38–41]. In particular, the Vmax value of *Aspergillus* phytase was almost identical to those of Bt and AC 1–2 MINPP1 (Table 3). In addition, the catalytic efficiency (Vmax/Km) of avian MINPP1 was 4-fold higher than that of *Bacillus licheniformis* PB-13 phytase [42].

### Substrate specificity

The substrate specificity of avian and AC 1–2 MINPP1 for phytate was strict because the hydrolysis for p-nitrophenyl phosphate (pNPP), a universal acid phosphatase substrate was less than 26% of the phytase activity [17,21]. Activities of pNPP and other phosphorylated conjugates by phypA and phylA MINPP1 were below 7% of phytase activity [22], showing highly strict substrate specificity. In particular, avian MINPP1 was more effective in hydrolyzing a physiologically genuine phytate pentamagnesium salt (Mg-InsP_6_) than commercial *Aspergillus niger* (*ficuum*) phytase [17]. Meanwhile, phosphatase activities for pNPP in barley and wheat MINPP1 were about 50% of the phytase activity [19]. These enzymes can hydrolyze ortho-carboxyphenyl phosphate with the same level as phytate [19], thus showing relatively broad substrate specificity. In this regard, it has been recently reported that wheat MINPP1 can degrade phosphorylated inflammatory substrates such as lipopolysaccharide (LPS) and inorganic polyphosphates (poly P) [43,44].

### Positional specificity

Most of the commercially available microbial phytases such as *Aspergillus niger* and *Bacillus* sp. are known as 3-phytases (EC 3.1.3.8) for catalyzing the initial release of phosphate linked at the 3’-position on the six carbon ring structure of phytate, while *E. coli* has a 6-phytase (EC 3.1.3.26) activity [21,41]. However, plant, animal, and bacterial MINPP1 including phypA, phylA, AC 1–2, and Bt lack the positional dephosphorylation specificity [19–23,45,46].

### Effect of metal ions on the enzyme activity

Phytase activities of wheat and barley MINPP1 are severely inhibited in the presence of Cu^2+^ or Zn^2+^ [19]. Low concentration (1 mM) of Ca^2+^ can act as an inhibitor for these enzymes [19]. In contrast, AC 1–2 MINPP1 is unaffected by the addition of Zn^2+^ [21], known as the strongest inhibitor of commercial *Aspergillus* phytase [47]. The phytase activity is stimulated in the presence of 1 mM Ca^2+^ [21], which has been reported as a stabilizing agent in catalytic functions of β–propeller *Bacillus* phytase [41]. In the case of phypA and phylA MINPP1, 2 mM of Ca^2+^ can stimulate phylA, but not phypA [22]. However, both enzymes are inhibited by higher concentrations (over 4 mM) of Ca^2+^ [22].

### Stability

Both phypA and phylA MINPP1 have good thermal stabilities with 73% and 44% of their initial activities retained at 80°C after 15 min, respectively [22]. Storage stability at room temperature is one of important factors when evaluating the quality of a commercial phytase product [48]. In some cases, the symptom of rickets in pigs is associated with poor inventory management of phytase after a long-term storage for 6 to 12 months [48]. According to Sulabo et al [48], six commercially available phytases maintained about 70% of their initial activities after storage at room temperature for 120 days. Meanwhile, the AC 1–2 MINPP1 was extremely stable after more than three years of storage [21].

## BIOACTIVE FUNCTIONS OF MINPP1

### A potential modifier for milk fatty acid traits in dairy cattle

Genome-wide association studies such as single nucleotide polymorphism have suggested that *MINPP1* may modulate the composition of milk fatty acids in dairy cattle [49]. For example, Chinese Holstein cows with g. 9207070A>G bearing the intron 5 mutation of *MINPP1* show a significant increase of C6:0 (hexanoic acid) but a significant decrease of C17:0 (heptadecanoic acid) [49]. Previously, intron mutation has been reported to be involved in developmental and cell-specific expression and over-expression of certain genes [50,51]. Presumably, MINPP1can affect on fatty acid related-metabolism by up-regulating the release of 2-phosphoglycerate from 2,3-bisphosphorglycerate, a negative allosteric regulator of hemoglobin in the unusual Rapport-Luebering glycolytic shunt, resulting in bypassing 3-phosphoglycerate of the common glycolysis [18], which can stimulate AMP-activated protein kinase (AMPK) signaling involved in the elevation of fatty acid oxidation [52]. Consequently, further investigation needs to validate the direct regulatory effect of *MINPP1* on compositional change of milk fatty acids in dairy cattle by knock-out, RNA interference, or gene editing approach [49].

### A potential additive for improving animal health

Wheat MINPP1 is effective in dephosphorylating LPS and long-chain inorganic polyphosphate (polyP) [43,44]. These two substrates are closely associated with sectors of animal husbandry. The former is a well-known pathogen-associated molecular pattern (PAMP). It is an endotoxin with structurally-phosphorylated toxic lipid A moiety borne from Gram-negative pathogenic bacteria and easily detectable as suspended dusts in swine and poultry farms [43]. The latter is a linear compound with about 200 to 1,000 phosphate residues linked by phosphodiester bond. It can act as a virulent factor in enteric disease-inducing bacteria including *Salmonella typhimurium* and *Campylobacter jejuni* frequently found in contaminated animal-resource foods [44,53].

According to An and Cho [43], LPS treated with wheat MINPP1 can significantly repress the release of interleukin (IL)-8, a universal pro-inflammatory cytokine, in HT-29 (an intestinal epithelial cell line) and reduce the cytotoxicity in HAE aortic endothelial cells. Long-chain polyP dephosphorylated by MINPP1 can also inhibit the secretion of IL-8 in HT-29 by deactivating nuclear factor-kappa B (NF-*k*B) [54]. Moreover, long-chain polyP digested with MINPP1 can improve nitric oxide (NO) production and *in vitro* phagocytic activity in Raw 264.7, a murine macrophage cell line [53]. Taken together, it appears that wheat MINPP1 functions as an immune-modulatory agent.

Another study has revealed that the exosome-enveloped human MINPP1 isoform-2 can function as a stimulator of tumor cell growth in a human breast cancer cell line, MCF-7 under cellular stress induced by brefeldin A, a known pharmacological protein trafficking inhibitor [29]. Human MINPP1 is known as an ER-based stress responder [30]. Under long-term temperature stress, common carp (*Cyprinus carpio*) *MINPP1* was one of the target genes involved in the regulation of glycolysis by the action of down-regulated micro RNAs (mi RNAs) [55] closely associated with heat stress in some vertebrates such as mice and Holstein cows [56,57]. As a stress alleviator, wheat MINPP1 can enhance cell viability by releasing IL-8 or IL-6 in a human colorectal adenocarcinoma cell line, HT-29 exposed to inflammatory nucleotides such as adenosine triphosphate (ATP) and uridine diphosphate (UDP), suggesting that MINPP1 might be a modulator of the growth and functions of intestinal epithelial cells under luminal ATP and UDP stimuli in the gut [58].

### A potential regulator of bone-related physiology

Chondrocyte differentiation is a main cellular event of skeletogenesis [27]. During the chondrocyte developmental process in some animals such as mouse, rat, chick and rabbit, it has been reported that *MINPP*1 expression is detectable in chondrocytes transiting from proliferation to hypertrophy [25,27,28]. In this regard, mechanisms on the catalytic function of MINPP1 remain puzzling since cellular inositol pentaphosphate (InsP_5_) and InsP_6_ pools as main substrates of the enzyme show no substantial changes in a mouse chondrogenic ATDC5 cell line with MINPP1 stably over-expressed [27]. Rather, the cell line overexpressing MINPP1 was defect of chondrogenesis [27]. Even *MINPP*1 knock-out mice have shown normal chondrocyte differentiation and bone development [59]. On the other hand, MINPP1 has been identified as one of suggestive genetic correlation signals in hand osteoarthritis (OA) using a human plasma proteome-based approach [60], although its functional role in OA development is poorly understood.

## CONCLUDING REMARKS

In terms of catalytic properties, MINPP1 is a promising alternative for phytate degradation. It can be compared to conventional microbial phytase. Still, soil and gut microbiomes are attractive sources for exploring a novel MINPP1 producer [61]. Recent advances on functional metagenomics fused with classical enzyme engineering and recombinant DNA technology can accelerate to develop MINPP1 as an ideal phytase [62].

MINPP1 can act as an anti-inflammatory agent by repressing IL-8 secretion under LPS and inorganic polyphosphate-induced stimuli, and improve the viability of the intestinal epithelial cells exposed to extracellular nucleotides such as ATP and UDP [43,54,58]. The MINPP1 gene appears to be a genetic marker in bone and OA development [59,60], and a molecular target for the modulation of milk fatty acid composition [49]. Taken together, MINPP1 may work favorably for the production performance of farm animals. Profound studies revealing the direct effect of MINPP1 on these physiological events are needed in the future.

## Figures and Tables

**Table 1:** The outline of the most recent positive results beyond the nutritional aspect on supplemental phytase in poultry and pig

Animal	Action mode	Enzyme source	Ref.
Broiler chickens	Inhibition of harmful bacteria including *Pelomonas*, *Helicobacter*, and *Pseudomonas*	VTR Biotech (2,000 FTU/kg diet)	[5]
Broiler chickens	Inhibition of harmful bacteria including *Escherichia coli* and *Clostridium* spp.	BASF (500 FTU/kg)	[6]
Broiler chickens	Elevation of GSH-Px, CAT, SOD, and total antioxidant capacity Reduction in serum MDA	Meriden Animal Health (5,000 FTU/kg)	[7]
Nursery pigs	Inhibition of harmful bacteria including *Prevotellaceae*	CJ Bio (2,000 FTU/kg)	[8]
Nursery pigs	Decrease of the plasma MDA	Quantum Blue AB Vista (500 FTU/kg)	[9]
Growing-finishing pigs	Inhibition of harmful bacteria including *Tenericutes* and *Spirochaetes*	Wuhan Sunhy Biology (500 U/kg)	[10]
Growing-finising pigs	Decrease of IL-1β and TNF-α release in the jejunal mucosa Elevation of GSH-Px and CAT activities in the duodenum and ileum	Challenge International Trade (1,000 U/kg)	[11]

GSH-Px, glutathione peroxidase; CAT, catalase; SOD, superoxide dismutase; MDA, malondialdehyde; IL-1β, interleukin-1 beta; TNF-α, tumor-necrosis factor-alpha.

**Table 2:** The summary of the representative physiological studies on MINPP1 since the year 2015

Species	Expected effect	Ref.
Human, mouse	Regulation of human brain development and homeostasis	[31,32]
Human, mouse	Regulation of ER stress and apoptosis	[30]
Human	Down-regulation in HBV-positive HCC and repressing the proliferation and migration of the tumor cells	[33]
Dairy cattle	Potential SNP candidate marker for milk fatty acid traits	[49]
Broiler chickens	Regulation of *myo*-Ins metabolism in blood and feather	[34]
Common carp	Up-regulatory candidate gene in glucose metabolism under temperature stress	[55]

ER, endoplasmic reticulum; HBV, hepatitis B virus; HCC, hepatocellular carcinoma; SNP, single nucleotide polymorphism; *myo*-Ins, *myo*-inositol.

**Table 3:** Enzymatic properties characterized by the known MINPP1s for phytate

Sources	Km (μM)	Vmax (μmol/min/mg)	Optimum pH	Optimum temperature (°C)	Mol. mass (kDa) estimated	Ref.
Avian	140	0.715	5–6 7.5	-	48	[17]
Human	90	0.0062	-	-	55	[17,18]
Wheat	246–393	-	4.5	65	54	[19]
Barley	334–517	-	4.5	65	54	[19]
*Bacteroides thetaiotaomicron* (Bt)	18.4	178	2.5 4 7.5	55	49	[20]
*Acinetobacter* sp. (AC1–2)	650	228	3 4.5–5 6	-	51	[21]
*Bifidobacterium pseudocatenulatum* (PhypA)	-	-	5.5	50	-	[22]
*Bifidobacterium longum* subsp. *infantis* (PhylA)	-	-	5.5	50	-	[22]

-, not available.
